# Effect of High Pressure Treatment on Poly(lactic acid)/Nano–TiO_2_ Composite Films

**DOI:** 10.3390/molecules23102621

**Published:** 2018-10-12

**Authors:** Hai Chi, Wenhui Li, Chunli Fan, Cheng Zhang, Lin Li, Yuyue Qin, Minglong Yuan

**Affiliations:** 1 Institute of Yunnan Food Safety, Kunming University of Science and Technology, Kunming 650550, China; 18468273140@163.com (H.C.); 15559823733@163.com (W.L.); fanchunli1995@163.com (C.F.); 13136640259@163.com (C.Z.); 2 College of Food Sciences and Engineering, South China University of Technology, Guangzhou 510640, China; felinli@scut.edu.cn; 3 Engineering Research Center of Biopolymer Functional Materials of Yunnan, Yunnan Minzu University, Kunming 650500, China

**Keywords:** poly(lactic acid), nano-TiO_2_, high pressure, composite film

## Abstract

The microstructure, thermal properties, mechanical properties and oxygen and water vapor barrier properties of a poly(lactic acid) (PLA)/nano-TiO_2_ composite film before and after high pressure treatment were studied. Structural analysis showed that the functional group structure of the high pressure treated composite film did not change. It was found that the high pressure treatment did not form new chemical bonds between the nanoparticles and the PLA. The micro-section of the composite film after high pressure treatment became very rough, and the structure was depressed. Through the analysis of thermal and mechanical properties, high pressure treatment can not only increase the strength and stiffness of the composite film, but also increase the crystallinity of the composite film. Through the analysis of barrier properties, it is found that the barrier properties of composite films after high pressure treatment were been improved by the applied high pressure treatment.

## 1. Introduction

In the past few decades, petroleum-based synthetic plastics have been widely used around the world, and a large number of industrial wastes generated by these polymers have been introduced to the ecosystem every year [1]. At present, the problem of environmental pollution is becoming more and more serious, therefore, more and more people are paying attention to biodegradable packaging materials.

Among many biodegradable materials, poly(lactic acid) (PLA), a starch derived from fermented plants originating from renewable resources, is rapidly becoming one of the alternatives to petroleum-based plastics [2,3]. As an environmentally friendly material, its performance is excellent and its use is extensive. It has immeasurable consumption potential and development prospects. Moreover, PLA-based packaging materials are considered safe (GRAS) [4], and in North America and Europe PLA has been used in bottles for water and fruit juices and milk packaging [5]. Although PLA has many advantages, it presents also some limitations such as a large brittleness and poor heat resistance. Meanwhile, its mechanical properties and gas barrier properties have yet to be further improved to meet the requirements of different applications. Plasticizers, polymers, and nanoparticles are frequently blended into PLA-based materials to improve the performance of PLA. The addition of these additives makes the PLA-based composites exhibit the corresponding biological activity and provides PLA with more diversified applications [6,7,8].

Recently, nanomaterials have been widely used in food packaging because they can be uniformly dispersed into polymer materials and they improve the performance of biodegradable polymers. One of the most commonly used nanoparticles in polymer composites is nanotitanium dioxide (nano-TiO_2_). Nano-TiO_2_ has high photosensitivity, low toxicity, antibacterial properties, strong oxidizability and long-term stability [9,10,11]. Therefore, the application of titanium dioxide plays a very important role in the modern food packaging field. For example, Xing et al. found that the addition of nano-TiO_2_ to polyethylene not only improved the performance of the polyethylene, but also greatly improved its antibacterial performance [12]. Nano-TiO_2_ has been approved by the U.S. Food and Drug Administration (FDA) for use in the food industry [13].

In recent years, high pressure technology has been used in the food industry, mainly to kill bacteria in foods to increase food safety and extend the shelf life of stored foods [14,15,16]. However, few people have studied the effects on the packaging film before and after high pressure treatment. Niu et al. found that the high pressure treatment can increase the tensile strength of chitosan films and lower the elongation percentage, water vapor permeability and oxygen transmission rate values. The structure of treated films is more stable and compact than that of control samples [17].

In this study, nanocomposites were prepared by solvent evaporation with PLA and different contents of nano-TiO_2_. The newly prepared nanocomposite film was treated with high pressure. The purpose of this study was to compare the effects of high pressure treatment on the properties of nanocomposite packaging films. The barrier properties of films (oxygen transmission rate (OTR) and water vapor permeability (WVP)), structural characterization, mechanics and thermal properties were measured.

## 2. Results and Discussion

### 2.1. FTIR

**Figure 1:**
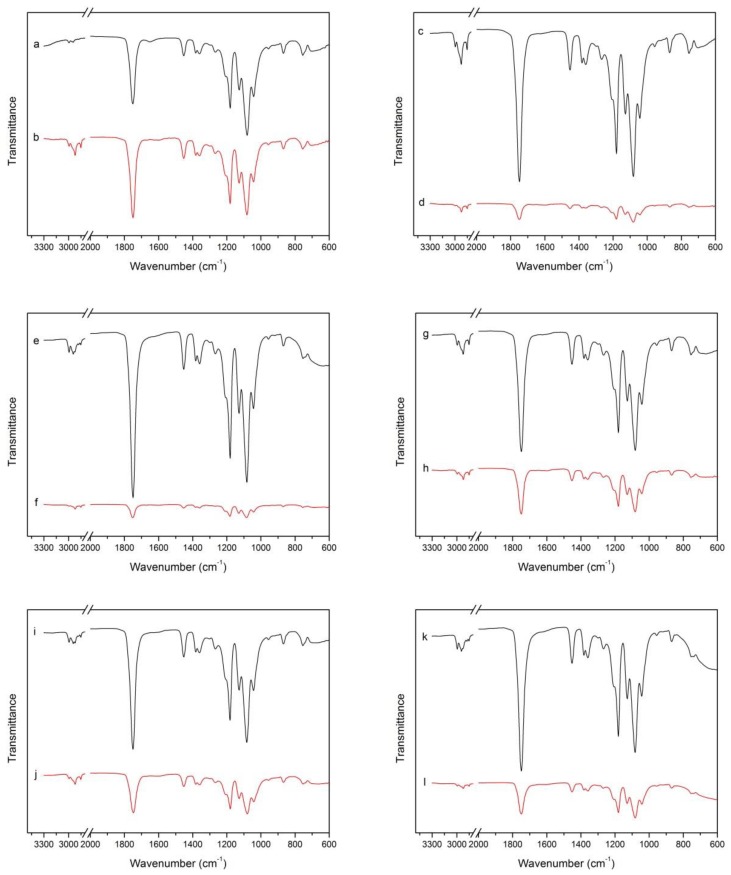
(**a**,**c**,**e**,**g**,**i**,**k**) represents the FTIR patterns of composite film PLA-0, PLA-1%, PLA-5%, PLA-10%, PLA-15% and PLA-20% before high pressure treatment, respectively; (**b**,**d**,**f**,**h**,**j**,**l**) represents the FTIR patterns of composite film PLA-0, PLA-1%, PLA-5%, PLA-10%, PLA-15% and PLA-20% after high pressure treatment, respectively.

FTIR spectra are sensitive to polymer structure changes at a molecular level. Figure 1 shows the FTIR spectra of PLA nanocomposite films with different nanoparticle contents before and after high pressure treatment, where it can be seen that the positions of the peaks of the spectra do not change.

For the spectrum of PLA composite films, the peak at 2994.5 cm^−1^ was assigned to the asymmetric C-H stretching vibration of the –CH_3_ group, and that at 2977.9 cm^−1^ to the symmetric one. A broad and strong absorption band at 1746.7 cm^−1^ was attributed to the ester –C=O stretching vibration. The peaks at 1452.3 and 1382 cm^−1^ indicated asymmetric and symmetric –CH_3_ deformation vibrations, respectively. The peaks at 1180.1 and 1080.4 cm^−1^ corresponded to asymmetric C–O–C stretching vibrations. The C–CH_3_ stretching vibration was assigned to the peak at 1041.5 cm^−1^. The peak at 867.1 cm^−1^ was ascribed to the amorphous crystalline phase [18,19,20].

From Figure 1, for the pure PLA film, the infrared spectrum of the film before and after the high pressure has substantially no changes in the position and the intensity of the peaks, which indicates that the high pressure treatment does not change the characteristic signals of the pure PLA film. In Figure 1, it can be seen that with the addition of nanoparticles, the positions of the characteristic signals of the composite film as reflected by the infrared spectrum remains unchanged, but the intensity of the peaks changed. By comparing the infrared curves of the composite films a, c, e, g, i and k, it is seen that as the content of nanoparticles added to the PLA matrix increases, the intensity of the internal characteristic signal of the composite film increases. This phenomenon is related to the addition of nanoparticles, because the addition of nanoparticles increases the intensity of the characteristic signals inside the composite film [21], but the high pressure treatment causes the nanoparticles adhered to the PLA but not embedded in the PLA composite film to fall off, resulting in a weaker characteristic signal when scanned with an infrared spectrometer than before the high pressure treatment.

### 2.2. SEM

**Figure 2:**
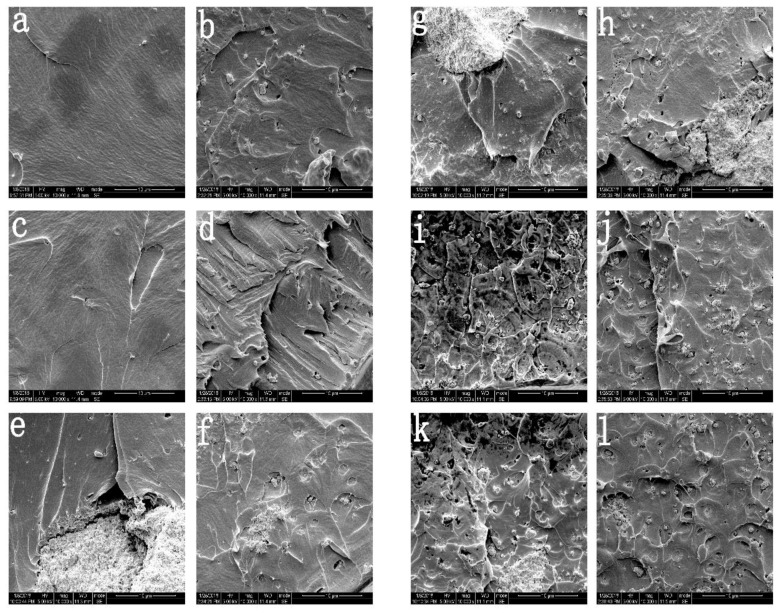
(**a**,**c**,**e**,**g**,**i**,**k**) represents the microstructure morphology of composite film PLA-0, PLA-1%, PLA-5%, PLA-10%, PLA-15% and PLA-20% before high pressure treatment, respectively (mag:10,000×); (**b**,**d**,**f**,**h**,**j**,**l**) represents the microstructure morphology of composite film PLA-0, PLA-1%, PLA-5%, PLA-10%, PLA-15% and PLA-20% after high pressure treatment, respectively (mag:10,000×).

In order to better understand the influence of high pressure treatment on the microsection morphology of the composite film materials, the microscopic morphology of the composite film was observed by scanning electron microscopy. By comparing the two graphs a and b in Figure 2, it can be found that the microscopic morphology of the neat PLA film is generally smooth and flat before high pressure application, and the PLA’s microscopic truncated surface showed uneven morphology after the high pressure treatment, and the overall microstructure showed a roughness phenomenon. The presence of a large amount of top air in the packaging film and the rapid depressurization can lead to uneven formation of the film. Fairclough et al. [14] found that after the high pressure treatment of PET/PP film, due to the presence of air, as the pressure increased, the gas was easily dissolved in the inner film. In the process of rapid pressure lowering, the rapid expansion and burst of the gas bubbles in the inner film resulted in the formation of sag.

As shown in Figure 2, with the increase of the content of nano-TiO_2_, it can be found that the influence on the microscopic cross-section morphology of the composite film before and after the high pressure treatment is getting smaller and smaller. Although there are still concave holes, they are less and less obvious. This phenomenon is related to the addition of TiO_2_. Through our previous research, it is known that the addition of nanoparticles will make the film form many micropores [22,23]. Therefore, the gas dissolves in the inner layer at low pressure during high pressure treatment, but when the pressure increases, the gas in the inner layer of the film will be extruded from the micro-pore by pressure, so it will not easily form a concave hole during the process of rapid pressure reduction, which explains why the effect of high pressure treatment on the composite film becomes smaller as the content of nanoparticles increases.

### 2.3. Mechanical Performances

Table 1 lists the effects of high pressure treatment on the mechanical properties (tensile strength (TS), elastic modulus (EM), and elongation at break (ε)) of nano-TiO_2_ composite films. As shown in the table, compared with the pure PLA film, in the nanoparticle composite film without high pressure treatment the TS value of the composite film increases first, and reaches the maximum TS value when the addition amount is 10% and then the TS value of the composite film decreases. This change in TS value may be due to the random and uniform dispersion of nanoparticles on the PLA matrix, which experiences interfacial adhesion under hydrogen bonding between the phases. In addition, as the content of nanoparticles increases, a large number of clusters are formed, so that the contact area between the nanoparticles and the PLA matrix is reduced, further reducing the TS value. For the EM value of the composite film, the change is similar to the TS value, and it also shows a tendency to increase first and then decrease. Compared with pure PLA film, the addition of nanoparticles can effectively improve the strength and rigidity of the composite film [24]. It can be seen from the table that when the composite film is subjected to pressure treatment, its TS value is increased. This is similar to our previous study, in which the formation of hydrogen bonds increased the stiffness of the composite film during high pressure processing [25]. In addition, the high-pressure treated nanocomposite film was analyzed by DSC and found to have higher crystallinity, which explained the increase of the EM value of the composite film after high pressure treatment. For the composite film ε value, the film without high pressure treatment has the opposite trend of TS and EM value, showing a trend of decreasing first and then increasing. The ε value of sample PLA-10 (72.1%) is the smallest, compared with pure PLA. The film was reduced by 13.9%. This trend may be due to the fact that nanoparticles limit the ductility of the PLA segment and may also be related to the aggregation of nanoparticles onto the PLA matrix. The high-pressure treated film has a significantly lower ε value than the non-high pressure treated film (*p* < 0.05), the elongation of the films decreased with the increase of crystallinity [26].

### 2.4. DSC

**Figure 3:**
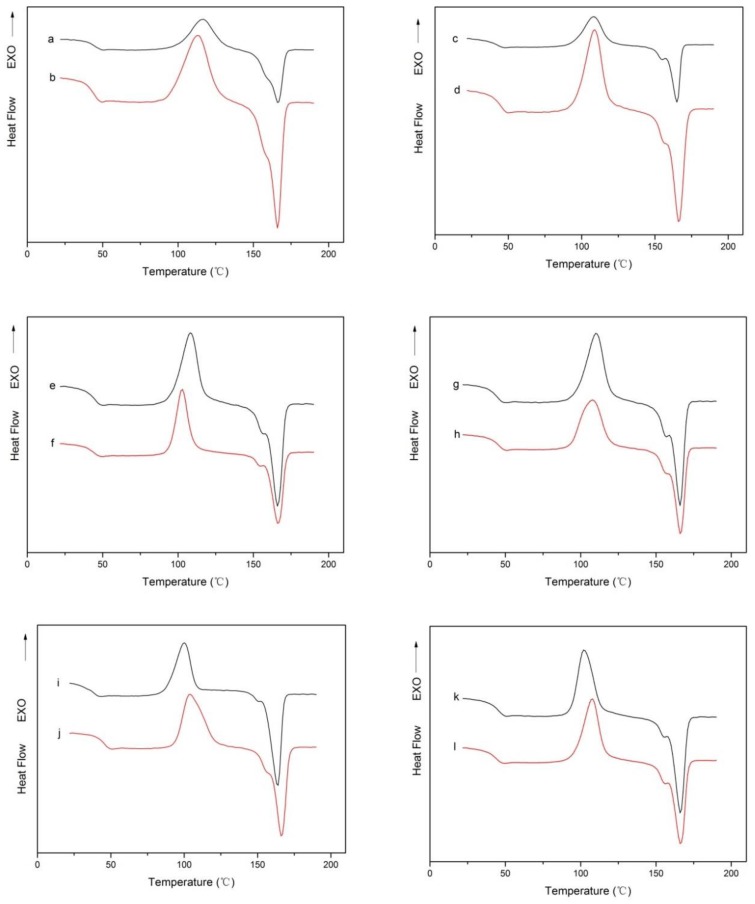
(**a**,**c**,**e**,**g**,**i**,**k**) represents the DSC curve of composite film PLA-0, PLA-1%, PLA-5%, PLA-10%, PLA-15% and PLA-20% before high pressure treatment, respectively; (**b**,**d**,**f**,**h**,**j**,**l**) represents the DSC curve of composite film PLA-0, PLA-1%, PLA-5%, PLA-10%, PLA-15% and PLA-20% after high pressure treatment, respectively.

Figure 3 shows the DSC curves of the nano-TiO_2_ composite film before and after high pressure treatment, and studies the effect of high pressure treatment on the thermodynamic properties of the composite film. Meanwhile, Table 2 lists the thermodynamic properties of the composite films obtained from the DSC curves, including the glass transition point (Tg), cold crystallization temperature (Tc), melting temperature [27] and the crystallinity (Xc) of the composite films.

It can be seen from the comparison of Figure 3 that the glass transition point (Tg), the cold crystallization temperature (Tc) and the melting temperature [27] of the composite film have not changed significantly before and after high pressure treatment. The crystallinity of the composite film was calculated according to the corresponding formula. From Table 2, it was found that the crystallinity of the composite film increased after high pressure treatment. This is because the pressure rise causes the amorphous region of the semi-crystalline thermoplastic polymer packaging material to change; the pressure rise also causes the temperature to rise, the temperature becomes larger, the segment movement of the amorphous region is enhanced, after unloading the chain motion is weakened, resulting in the molecular chain alignment close to form a crystalline state of the film.

Yoo et al. [28] found through studies that the crystallinity of polymers is affected by temperature and pressure, and increases with temperature and pressure. Caner et al. [29] performed pressure treatment (600 MPa, 25 °C, 10 min) on three films, such as met-PET/PE, and compared their condensation and melting temperatures. The results showed that the condensation and melting temperatures of the three kinds of films were not affected by high pressure treatment.

### 2.5. Water Vapor Permeability (WVP)

Water vapor plays an unparalleled role in food spoilage and microbial growth, reducing or even preventing water vapor from entering the internal environment through the food packaging bag, which affects the flavor and taste of the food. In other words, water vapor transmission rate (WVP) has become an important performance indicator for food packaging materials.

From Table 3, we can see that with the increase of nanoparticles, the overall trend of water permeability of composite film is firstly reduced and then increased, which is also in line with our previous experiments [22]. The reason for this trend is that when low-concentration nano-TiO_2_ is added to the PLA matrix, the nanoparticles will be embedded into the PLA matrix. In this way, when water vapor passes through the composite film, the path of water vapor through the composite film becomes longer due to the presence of nanoparticles, so the water vapor transmission rate decreases. Then, as the nanoparticles increase, aggregation occurs in the PLA matrix to form clusters, which cause the composite film to form a large number of pores, resulting in an increase in water vapor transmission rate [30,31].

Comparing the water vapor transmission rate of the composite film before and after the high pressure, it is found that the high pressure treatment will reduce the water permeability of the composite film, which may be related to the crystallinity change in the composite film. It can be seen from Table 2 that the high pressure treatment increases the crystallinity of the composite film, and the inside of the composite film is more closely arranged, thereby causing a decrease in water vapor transmission performance of the composite film.

### 2.6. Oxygen Transmission Rate (OTR)

The oxygen transmission rate of the composite film before and after the high pressure treatment is shown in Table 4. It can be seen from the chart that the OTR value of the composite film decreases with the increase of nano-TiO_2_ content. Among them, pure PLA had the highest OTR value, and PLA-5% had the lowest OTR value, which decreased 25.5%. The decrease of OTR value exactly indicates the improvement of oxygen barrier ability of composite film. This improvement in barrier properties may be due to the uneven dispersion of nano-TiO_2_, which results in a strong interfacial adsorption between the PLA matrix and the nanoparticles, which further leads to the immobilization of the PLA segment and reduces the diffusion of substances in the PLA matrix [32]. In addition, the increase in oxygen barrier capacity of the composite film may be related to the crystallinity of the composite film. However, when the nano-TiO_2_ content is increased to 10 wt%, the OTR value of the composite film will no longer decrease but increase significantly. This may be due to the aggregation of nanoparticles on the PLA matrix, resulting in the initialization of aggregated particles and the formation of osmotic channels, making the gas pass faster, the barrier performance dramatically decreased.

Comparing the OTR values of the composite film before and after the high pressure treatment, it was found that the high pressure treatment reduced the OTR of the composite film. The improvement of the oxygen barrier property of the composite film may be related to the crystallinity of the composite film. As shown in Table 2, the high pressure treatment increases the crystallinity, and the structure of the composite film is compressed, thereby reducing the oxygen transmission rate.

## 3. Materials and Methods

### 3.1. Materials

PLA (Mw = 280 kDa, Mw/Mn = 1.98) was obtained from Natureworks LLC (Blair, NE, USA). Nano-TiO_2_ used in this study was purchased from Jinda Nano Tech Co., Ltd. (Xiamen, Fujian, China) with an average particle size <100 nm and purity of 99.5%. Acetyltributyl citrate (ATBC) was purchased from Chou Feng Enterprise Co., Ltd. (Shulin, Taiwan). Dichloromethane was obtained from Chengdu Kelong Chemical Co., Ltd. (Chengdu, China).

### 3.2. Preparation of Nanocomposites

The films used in this study were prepared by solvent evaporation method. Before preparing the film solution, the PLA particles were placed in the oven at 60 °C for 10 h to eliminate the influence of moisture. PLA (2 h), ATBC plasticizer and different percentages (1%, 5%, 10%, 15%, 20%) of the nano-TiO_2_ were weighed accurately and mixed with dichloromethane (50 mL0 in a conical flask. Then the mixture was stirred for 8 h at room temperature with a magnetic stirrer until the film forming liquid is completely mixed, and then the flask was placed under ultrasonic irradiation or 1 h under constant temperature to uniformly disperse the nanoparticles in the PLA matrix. Finally, the film forming liquid is evenly poured into the glass plate (20 cm × 20 cm). The composite film can be obtained by placing the glass plate in a dry environment and placing overnight. These composite films were named PLA-0, PLA-1%, PLA-5%, PLA-10%, PLA-15%, PLA-20%. PLA-0 which does included added nano-TiO_2_ powder, used as a control.

The composite film is placed in a vacuum bag to avoid contamination by the medium water in the high pressure processing equipment. After the vacuuming treatment, the sealed bag was placed in a sample chamber of a high-pressure treatment apparatus, and treated under a pressure of 300 MPa for 10 min.

### 3.3. Fourier Transform Infrared

Infrared spectroscopic analysis was performed using a Nicolet-5700 FTIR spectrometer (Ametek, Inc., Shanghai, China). The tested spectral range was 400 cm^−1^ to 4000 cm^−1^ with a resolution of 4 cm^−1^.

### 3.4. Scanning Electron Microscopy

A Nano SEM 450 scanning electron microscope (NOVA, Chengdu, China) was used to observe the surface topography of the sample at a scanning voltage of 10 kV. Before observation, the sample was placed in liquid nitrogen for natural fracture treatment and the surface gold treatment sprayed. 

### 3.5. Mechanical Performances

Mechanical properties including elongation at break (ε), tensile strength (TS) and elastic modulus (EM) [33] were measured by a universal stretcher (CMT 4104, MTS Systems Co., Ltd., Shenzhen, China). Before the test, the samples were cut into 15 mm × 100 mm long strips. According to ASTM d882-88, the tensile speed is 50 mm/min. For each sample, we took five measurements and report the average.

### 3.6. Differential Scanning Calorimetry

Thermodynamic characterization was performed using a DSC 214 instrument (Netzsch, Selb, Germany) under pure nitrogen. First of all, a weighed sample (10 mg) in a sealed aluminum crucible, was heated from 10 °C to 200 °C at 10 °C/min speed, and then held for 5 min to eliminate thermal history, and then cooled to 10 °C at a rate of 20 °C/min, and then heated at 10 °C/min to 200 °C to obtain the glass transition temperature (Tg), the melting temperature [27] and the cold crystallization temperature (Tc). In addition, the percentage of crystallinity (Xc) is calculated according to the following formula [34]:

(1) $$Xc(\%)=(\Delta Hm/\Delta H_{m}^{0}w)\times 100$$

where Δ*H_m_* is the enthalpy of melting (J/g), $\Delta H_{m}^{0}$ is the heat of fusion for completely crystalline PLA (93.7 J/g), and w is the weight fraction of PLA in the samples.

### 3.7. Water Vapor Permeability

The water vapor permeability (WVP) of films was tested with the ASTM e96-95 standard method [27]. Discolored silica gel (12 g) was accurately weighed into a weighing bottle, tightly covered with the composite film that was fixed on the weighing bottle with a leather band and sealing film, then weighed, and placed in the bottom of a desiccator filled with saturated NaCl solution. Finally, the desiccator is placed in an environment at a temperature of 25 °C and a relative humidity of 65% for 24 h and weighed at a given time. The water vapor transmission rate is calculated according to the following formula:

(2) $$\mathrm{WVP}=\frac{\Delta w\times X}{\Delta t\times A\times \Delta P}$$

where Δ*w* is the change in weight of the weighing bottle (g), *X* is the average film thickness (m), Δ*t* is the measured time (s), *A* is the area of the test area (m^2^), Δ*P* is the difference in water vapor pressure across the films.

### 3.8. Oxygen Transmission Rate

In this study, we have used an oxygen transmittance analyzer (Oxy Sense 5250i oxygen analyzer, OxySense, Inc., New Castle, DE, USA) to measure the Oxygen transmission rate (OTR) value. The film was cut into circles of 5 cm in diameter and tightly adhered with a sealant to a sample chamber at an environment temperature of 25 °C. Pure nitrogen was introduced into the upper part of the sample chamber first to replace air, and then, pure oxygen is introduced into the lower half of the sample chamber for measurement. Multiplying the thickness of the films with the OTR value and dividing it by the pressure difference in the chamber, finally we get the permeability [35]. Each film is measured three times, taking its mean value.

## 4. Conclusions

In this study, nano-TiO_2_ was embedded into PLA by melt blending to form PLA-based nano-TiO_2_ composites, and then the composite film materials were treated under high pressure. Then the properties of the composite film before and after high pressure treatment were tested and compared. It was found by FTIR test that the functional group structure of the composite film did not change after high pressure treatment. The microscopic brittle fracture cross-section of the composite film was observed by scanning electron microscopy. It was found that the microsection of the composite film without high pressure treatment was relatively flat, and the composite film after high pressure treatment appeared rougher and many depressions appeared. The mechanical properties analysis found that the high pressure treatment can improve the stiffness and strength of the nanocomposite film. It was found by DSC that the composite films before and after high pressure treatment had no significant change in other thermodynamic properties except for the crystallinity of the composite film. By measuring the barrier properties of the composite films, it was found that the water vapor permeability and oxygen transmission rate of the composite films decreased with the increase of the crystallinity of the composite films after high pressure treatment. This finding is important for the development of safe food packaging materials with higher properties.

## Figures and Tables

**Table 1:** The mechanical properties of composite films.

AgNPs (wt%)	Pressure (MPa)	TS (MPa)	EM (MPa)	ε (%)
0	0	30.71 ± 1.18 ^b^	1265.4 ± 71.3 ^e^	83.7 ± 5.21 ^a^
0	300	31.32 ±0.94^a,b^	1327.3 ± 82.2 ^d,e^	79.1 ± 5.86 ^a,b^
1	0	32.71 ± 1.47 ^a,b^	1455.5 ± 56.3 ^c,d,e^	78.2 ± 6.51 ^a,b^
1	300	33.85 ± 2.41 ^a,b^	1515.3 ± 96.9 ^b,c,d,e^	70.6 ± 7.32 ^a,b,c^
5	0	33.50 ± 1.16 ^a,b^	1669.9 ± 87.7 ^a,b,c^	75.9 ± 5.58 ^a,b,c^
5	300	34.74 ± 1.47 ^a,b^	1841.5 ± 72.6 ^a^	67.9 ± 6.59 ^b,c^
10	0	34.89 ± 1.19 ^a,b^	1723.5 ± 85.3 ^a,b,c^	72.1 ± 6.81 ^a,b,c^
10	300	36.08 ± 1.25 ^a,b^	1874.2 ± 90.2 ^a^	64.2 ± 4.62 ^c^
15	0	33.95 ± 1.75 ^a,b^	1692.1 ± 102.4 ^a,b,c^	73.3 ± 5.02 ^a,b,c^
15	300	36.17 ± 1.96 ^a^	1783.3 ± 103.3 ^a,b^	67.8 ± 4.88 ^b,c^
20	0	32.45 ± 1.42 ^a,b^	1596.2 ± 96.8 ^a,b,c,d^	75.2 ± 5.12 ^a,b,c^
20	300	33.72 ± 1.78 ^a,b^	1687.1 ± 88.2 ^a,b,c^	72.1 ± 5.67 ^a,b,c^

^a–e^ Values followed by different superscripts in the same column denote significant difference (*p* < 0.05), where a is the highest value.

**Table 2:** Thermodynamic characterization of composite films.

Samples (wt%)	Pressure (MPa)	Tg (°C)	Tc (°C)	Tm (°C)	Xc (%)
0	0	45.2	112.2	168.2	14.5
0	300	45.8	108.3	172.1	18.3
1	0	47.8	109.5	165.1	15.3
1	300	49.3	108.8	166.4	17.9
5	0	48.5	116.2	168.4	15.8
5	300	49.8	109.6	168.3	19.2
10	0	49.7	105.2	168.4	18.7
10	300	48.8	102.5	168.5	22.4
15	0	48.7	100.1	165.6	18.1
15	300	51.2	103.9	166.4	22.2
20	0	50.0	101.3	166.2	17.6
20	300	48.8	107.1	166.6	23.6

**Table 3:** Water vapor permeability of composite films with different ratios of nanoparticles before and after high pressure treatment.

TiO_2_ Content %	WVP (g·m)/(m^2^·s·Pa)	
	Before High Pressure Treatment	After High Pressure Treatment
0	5.28 ± 0.08 ^b^	4.81 + 0.17 ^a^
1	4.86 ± 0.14 ^b^	3.69 + 0.10 ^a^
5	4.22 ± 0.16 ^b^	3.46 + 0.32 ^a^
10	4.78 ± 0.26 ^b^	4.12 + 0.15 ^a^
15	5.13 ± 0.10 ^b^	4.40 + 0.12 ^a^
20	5.33 ± 0.17 ^b^	4.97 + 0.18 ^a^

^a,b^ Values followed by different superscripts in the same row denote significant difference (*p* < 0.05), where a is the lowest value.

**Table 4:** Oxygen Transmission Rate of composite films with different ratios of nanoparticles before and after high pressure treatment.

TiO_2_ Content %	OTR [(cm^3^/(24 h × m^2^)] × (cm/bar)	
	Before High Pressure Treatment	After High Pressure Treatment
0	4.39 ± 0.06 ^b^	4.02 +0.18 ^a^
1	3.82 ± 0.11 ^b^	3.53 + 0.12 ^a^
5	3.27 ± 0.14 ^b^	2.92 + 0.13 ^a^
10	3.56 ± 0.11 ^b^	3.28 + 0.09 ^a^
15	3.99 ± 0.14 ^b^	3.67 + 0.15 ^a^
20	4.30 ± 0.08 ^b^	3.98 + 0.21 ^a^

^a,b^ Values followed by different superscripts in the same row denote significant difference (*p* < 0.05), where a is the lowest value.
